# Ultra-High Field 7T MRI in a Drug-Resistant Pediatric Epilepsy Cohort: Image Comparison and Radiologic Outcomes

**DOI:** 10.1212/WNL.0000000000213921

**Published:** 2025-08-20

**Authors:** Katy Vecchiato, Chiara Casella, Ayse Sila Dokumaci, Ata Siddiqui, Alexia Egloff, Olivia Carney, Jon O. Cleary, Jozef Jarosz, Pierluigi Di Ciò, Michela Cleri, Kathleen Colford, Rory J. Piper, Tomoki Arichi, Michael Eyre, Fraser Aitken, Raphael Tomi-Tricot, Thomas Wilkinson, Joseph V. Hajnal, Colm J. McGinnity, Siti N. Yaakub, Sharon L. Giles, Shaihan Malik, Alexander Hammers, Philippa Bridgen, David W. Carmichael, Jonathan O'Muircheartaigh

**Affiliations:** 1 Department for Forensic and Neurodevelopmental Sciences, Institute of Psychiatry, Psychology and Neuroscience, King's College London, United Kingdom;; 2 Research Department of Early Life Imaging, School of Biomedical Engineering and Imaging Sciences, King's College London, United Kingdom;; 3 London Collaborative Ultra high field System (LoCUS), St Thomas' Hospital, United Kingdom;; 4 School of Biomedical Engineering and Imaging Sciences, King's College London, United Kingdom;; 5 Department of Radiology, Guy's and St. Thomas' NHS Foundation Trust, London, United Kingdom;; 6 Department of Neuroradiology, King's College Hospital NHS Foundation Trust, London, United Kingdom;; 7 Guys and St Thomas' NHS Foundation Trust, King's College London, United Kingdom;; 8 Developmental Neurosciences Research and Teaching Department, UCL Great Ormond Street Institute of Child Health, London, United Kingdom;; 9 Department of Neurosurgery, Great Ormond Street Hospital, London, United Kingdom;; 10 MRC Centre for Neurodevelopmental Disorders, King's College London, United Kingdom;; 11 Children's Neurosciences, Evelina London Children's Hospital at Guy's and St Thomas' NHS Foundation Trust, London, United Kingdom;; 12 MR Research Collaborations, Siemens Healthcare Limited, Camberley, United Kingdom; and; 13 King's College London & Guy's and St Thomas' PET Centre, School of Biomedical Engineering and Imaging Sciences, King's College London, United Kingdom.

## Abstract

**Background and Objectives:**

Epileptogenic lesions in focal epilepsy can be subtle or remain undetected on conventional MRI. Ultra-high field (7T) MRI offers higher spatial resolution, contrast, and signal-to-noise ratio compared with conventional field strengths and has shown promise in adult presurgical evaluation. However, its utility in pediatric focal epilepsy, where malformations of cortical development are common, remains unclear. In this study, we directly compared 7T and 3T MRI in children with drug-resistant focal epilepsy, evaluating (1) scan tolerability, (2) image quality, and (3) lesion detection yield.

**Methods:**

In this prospective cohort study, patients were recruited from outpatient clinics at 3 large epilepsy centers in London. Eligible participants were aged 8–17 years and had no contraindications to MRI. Scanning was conducted in 2 separate sessions at 3T and 7T. Safety and tolerability were assessed using age-specific questionnaires and analyzed using the Mann-Whitney test for differences between age groups and the McNemar χ^2^ test for within-participant cross-field comparisons. Image quality was evaluated by a pediatric neuroradiologist and estimated quantitatively by comparing cortical thickness between field strengths. Lesion detection yield of 7T MRI was assessed by joint review with a multidisciplinary team.

**Results:**

A total of 63 children were assessed: 41 patients (mean age 12.6 ± 2.4 years, 22 were male) and 22 healthy controls (8–17 years, mean age 11.7 ± 2.7 years, 15 were male). Both field strengths were well tolerated, and side effects were transient. These included higher dizziness-related discomfort at 7T (*p* = 0.02, Cohen *h* = 0.89), with side effects more frequently noted in younger children (scanner noise: *p* = 0.02, Cohen *r* = −0.36; metallic taste: *p* = 0.02, Cohen *r* = −0.37). Cortical thickness was significantly thinner at 7T for both right (*t* = 5.65, *p* < 0.001, Cohen *d* = −0.72) and left (*t* = 5.01, *p* < 0.001, Cohen *d* = −0.64) hemispheres, with thinner boundaries in temporo-parietal/sensory regions. Although 7T images had increased reported inhomogeneity and artifacts, new lesions were detected in 6 of 26 patients (23%), influencing surgical management in 4 of 26 (15%) (odds ratio 1.86).

**Discussion:**

7T MRI in children with epilepsy is well tolerated and associated with a 23% improvement in lesion detection, directly affecting clinical management and surgical outcomes. Although limited by a preselected cohort with extensive diagnostic workups, our findings underscore the transformative potential of 7T MRI in presurgical planning for pediatric epilepsy.

## Introduction

Epilepsy is a chronic neurologic condition that profoundly affects health and quality of life.^1^ Approximately 40% of patients with focal epilepsy are drug-resistant.^2^ In around two-thirds of patients, resection of epileptogenic tissue can lead to seizure freedom, or a marked (>90%) reduction in seizures,^3,4^ as well as potential improvements in cognitive and developmental outcomes.^5^

Although the identification of a lesion on imaging is the single biggest predictor of surgical outcome,^6-8^ this can be challenging in children. Conventional (1.5T or 3T) brain MRI can be used to identify structural epileptogenic abnormalities; however, approximately 30% of all patients with focal epilepsy (both adults and children) present with no findings on MRI (MRI negative).^5-8^ This is especially true for focal cortical dysplasias (FCDs) and other malformations of cortical development, which can be small and visually subtle in radiologic appearance^9^ and are more common in children.^10^ Failure to detect a structural lesion on MRI adds complexity to neurosurgical planning, can lead to further invasive investigations, and is associated with a less favorable prognosis.^11^

PET can help localize occult focal abnormalities by highlighting alterations in focal metabolism,^12,13^ and its diagnostic sensitivity is further improved after co-registration with MRI.^14^ However, the area of hypometabolism identified with PET scans often extends beyond the epileptogenic zone; thus, this technique cannot precisely define the lesion surgical margins.^15^

Ultra-high field (7T) MRI offers higher signal-to-noise ratio (SNR) and contrast compared with conventional MRI, enabling higher spatial resolution. This translates into a clearer delineation of anatomical structures, potentially increasing radiologic detection and improving diagnostic confidence.^16^ Previous studies have demonstrated that 7T MRI significantly improves lesion detection and clinical decision making in adults with epilepsy, revealing additional lesions and enhancing diagnostic confidence,^17^ with a diagnostic gain over conventional MRI ranging between 22% and 43%.^18-22^ However, the clinical efficacy of 7T MRI for lesion detection in children is not known. Moreover, there is no direct comparison of the performance of 7T MRI with clinically available 3T MRI in this clinical population.

It is important to note that a comprehensive understanding of the practical utility of 7T MRI in children hinges on recognizing its associated side effects and how it is perceived during examinations. While studies in adults have consistently demonstrated that discomfort is transient and 7T scans are generally well tolerated,^23,24^ the experience may vary in children because of age-related factors and developmental stages.

In this study, we present findings from a large prospective pediatric case series using 7T MRI with a paired 3T acquisition, to investigate the relative tolerability, image quality, and clinical yield of 7T MRI in children with drug-resistant focal epilepsy.

## Methods

### Participants

**Figure 1:**
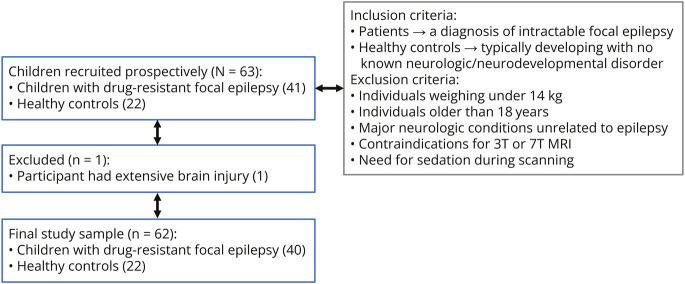
Flow Diagram Inclusion/exclusion criteria, initial number of participants, and those excluded from the study are shown. Intractable = failure of adequate trials of 2 tolerated and appropriately chosen antiepileptic drugs.

Patients were identified from epilepsy outpatient clinics at Evelina London Children's Hospital, King's College Hospital, and Great Ormond Street Hospital. Inclusion was based on confirmed diagnoses of epilepsy, with a specific focus on children and adolescents. The aim of this study was to include consecutive patients meeting the inclusion criteria to minimize bias and ensure a comprehensive representation of the population. Healthy controls with no history of neurologic or neurodevelopmental disorders were recruited using several strategies, including reaching out to families already enrolled in volunteer databases, contacting schools and community programs, and engaging parents whose children had previously participated in research. These controls were not family members of the patients but were identified and recruited from the broader community to serve as an appropriate comparison group. Inclusion and exclusion criteria for the study are presented in Figure 1. In addition to previous MRI data, patients' clinical information and previous investigations were accessible. PET scans were available in 29 of 41 patients, which were co-registered with the MRI scans as part of clinical reporting.^25^ However, this was not an explicit inclusion criterion.

### Data Acquisition

Data were collected between November 2019 and June 2023. All participants underwent 3T MRI including magnetization-prepared rapid gradient-echo (MPRAGE), fluid-attenuated inversion recovery (FLAIR), and T2-weighted sequences. Scans were acquired using the distributed and incoherent sample orders for reconstruction deblurring using encoding redundancy (DISORDER) scheme,^26^ which has demonstrated improved tolerance against motion.^27^ In-depth detail of the reconstruction algorithm has been described previously.^26,27^ Participants also underwent 7T MRI, including 3D magnetization-prepared 2 rapid acquisition gradient-echo (MP2RAGE),^28^ 3D FLAIR, and 2D turbo spin-echo T2-weighted sequences.^29^ Acquisition parameters are given in Table 1.

**Table 1:** Acquisition Details

Scanner	Sequence	TR/TE (msec)	Resolution	Acquisition time
3T	MPRAGE	7.7/3.6	1 mm isotropic	4 min 46 s
	FLAIR	5,000/422	1 mm isotropic	8 min 30 s
	T2-weighted	2,500/344	1 mm isotropic	5 min 42 s
7T	MP2RAGE^28^	4,000/3.15	0.65 mm isotropic	7 min 18 s
	1Tx FLAIR	9,000/240	0.8 mm isotropic	7 min 5 s
	PTx FLAIR	9,000/325	0.8 mm isotropic	6 min 54 s
	2D TSE T2-weighted (axial, coronal oblique, sagittal)^29^	8,100/82	0.4 × 0.4 × 2.0 mm	4 min 5 s each

Abbreviations: 1Tx = single RF transmission; FLAIR = fluid-attenuated inversion recovery; MPRAGE = magnetization-prepared rapid gradient-echo; MP2RAGE = magnetization-prepared 2 rapid acquisition gradient-echo; PTx = parallel RF transmission; TE = echo time; TR = repetition time.

The 3T scanning session was performed at Evelina Neuroimaging Centre on an Achieva 3.0T system (Philips Healthcare, Best, the Netherlands), whereas the 7T scan was performed in the London Collaborative Ultra-High Field System facility on a MAGNETOM Terra system (Siemens Healthineers, Erlangen, Germany). Both facilities are located at St Thomas' Hospital. The 2 scanning sessions were performed with a maximum interscan interval of 4 months. All participants were unsedated, wore earplugs for hearing protection, and watched a movie during scanning. Acquisition details are presented in Table 1.

**Figure 2:**
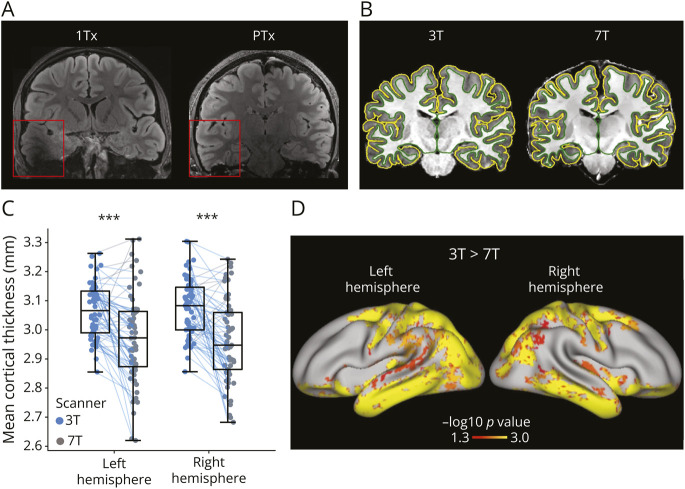
Imaging Characteristics of 3T and 7T MRI (A) Impact of parallel RF transmission on the FLAIR image of a healthy control. The image on the left, acquired with 1Tx, shows a visible signal drop in the right temporal lobe. No signal drop is present on the image on the right, acquired with PTx. (B) Example of a cortical delineation in a single participant at 3T and 7T. (C, D) Cortical thickness evaluation. Overall, the detected cortical thickness was thinner at 7T for both left (*t* = 5.01, *p* < 0.001) and right (*t* = 5.65, *p* < 0.001) hemispheres (C), with thinner boundaries in temporo-parietal/sensory regions (D). 1Tx = single RF transmission; FLAIR = fluid-attenuated inversion recovery; PTx = parallel RF transmission; RF = radiofrequency.

After the 21st participant, the 7T MRI protocol was adapted so that the FLAIR sequence was acquired using a parallel radiofrequency (RF) transmission (PTx) coil with the system switched to prototype research configuration, to correct for B_1_ field inhomogeneities affecting the right temporal lobe (Figure 2A). When scanning in the PTx mode, the FLAIR sequence from the PASTeUR package^30^ was used in 3D, as well as the DiSCoVER method^29^ for 2D T2-weighted imaging. Note that in line with local ethical approval, PTx was only available for subjects with mass 30 kg and over. Those with mass below 30 kg but above 14 kg were imaged using single channel transmission only. Notably, following the introduction of PTx, all participants met this weight criterion except for one patient, who was imaged using single-channel RF transmission due to a body mass below 30 kg.

### Analysis

#### Safety and Tolerability

A survey adapted from a previous 3T study in children^31^ was used to investigate general comfort and the presence of temporary collateral effects during 7T scanning in a subgroup of 45 participants (patients and controls) (eAppendix 1). Although the survey included several questions, we focused on the most reported side effects for meaningful comparison, specifically assessing discomfort, dizziness, noise, metallic taste, and body twitching.

Patients aged 8–11 and 12–17 years were given age-appropriate questionnaires and asked to rate the answers according to a 5-point Likert scale while for those aged 5–7 years, pictograms were used to explain those feelings and they were asked to respond with “yes” or “no.” Overall, 20 surveys were collected for the group aged 8–11 years, 20 for the group aged 12–17 years, and 3 for the group aged 5–7 years.

A Mann-Whitney test was conducted to analyze differences in tolerability and collateral effects at 7T between the groups aged 8–11 and 12–17. The youngest cohort sample size was too small for formal statistical testing and was excluded from the analysis.

In addition, to assess differences in tolerability between 3T and 7T, matched questionnaires were collected from a subgroup of eleven participants aged 8–16 years (mean age 13). The answers were dichotomized into “yes” (scores 1–3) and “no” (scores 4 and 5). Differences were tested using the McNemar χ^2^ test for dichotomized data.

#### Quantitative Assessment of Cortical Thickness

Preprocessing steps for 3T images included motion correction with DISORDER^26^ and Gibbs ringing correction.^32^ 7T MR images were denoised^33^ and bias field–corrected.^34^ Both 3T and 7T images were then processed with the Human Connectome Project (HCP) structural pipeline to perform cortical surface reconstruction and cortical thickness computation^35^ (Figure 2B). Both T1-weighted (3T: MPRAGE, 7T: denoised combined “UNI” image from the MP2RAGE acquisition) and T2-weighted (3T, 7T: FLAIR) scans were used for segmentation, the latter specifically to improve pial surface reconstruction.

Mean cortical thickness was extracted for both hemispheres, and a within-participant comparison was performed between 3T and 7T, investigating regional cortical thickness as a surrogate for improved delineation of the gray-white matter boundary. Statistical testing was performed on both mean cortical thickness using paired-sample *t* tests and cortical surface–based measurements, with permutation-based paired *t* tests implemented in the Permutation Analysis of Linear Models analysis package^36^ and threshold-free cluster enhancement.^37^ Age and sex were included as covariates. Correction for the family-wise error rate was applied across contrasts (contrast 1: 7T > 3T; contrast 2: 3T > 7T).

#### Radiologic Image Quality Assessment

To assess image visual quality, an expert pediatric neuroradiologist not involved in the clinical assessment reviewed the first 20 participants who underwent the 7T MRI, using a rating scale from the study by Wang et al.^38^ This expert was blinded to any demographic data, group identity, electrophysiology data, and PET information. They evaluated both 3T and 7T MRI images—comparing 3T MPRAGE with 7T MP2RAGE for T1-weighted contrast and 3T FLAIR with 7T FLAIR for T2-weighted contrast. The assessment included a subgroup of 10 patients (a mix of lesion-positive and lesion-negative) and 10 healthy controls. For 3T images, only motion-corrected versions were shown. The scoring was based on the presence of artifacts that could affect image quality and readability, such as motion, pulsation, and inhomogeneity.

#### Clinical Value of 7T MRI

The 3T and 7T images were reviewed by 3 expert neuroradiologists with longstanding experience in pediatric epilepsy imaging; the neurology, neurosurgery, and neurophysiology team; and, where relevant, a PET expert. Consensus in imaging interpretation was based on the identification of a structural abnormality that was concordant regarding focus location with electroclinical and, when available, PET data.

### Standard Protocol Approvals, Registrations, and Patient Consents

Ethical approval was granted by the UK Health Research Authority and Health and Care Research Wales (ethics ref. 18/LO/1766). Written signed consent was obtained from the parents (or the person with legal parental responsibility) before data collection.

### Data Availability

The clinical and neuroimaging data used in this study are available from the senior author (J.O.M.) on formal request indicating name and affiliation of the researcher as well as a brief description of the intended use of the data. All requests will undergo King's College London–regulated procedure, thus requiring submission of a Material Transfer Agreement. Full preprocessing steps and the code to run the HCP preprocessing pipeline can be found at github.com/Washington-University/HCPpipelines. the source code for Connectome Workbench is available at github.com/Washington-University/workbench. Other code excerpts, information regarding the analysis, or intermediary results can be made available on request to chiara.casella@kcl.ac.uk.

## Results

### Participants

A total of 63 children were recruited: 41 with drug-resistant focal epilepsy (8–17 years, mean age 12.6 ± 2.4 years, 22 were male) and 22 healthy controls (8–17 years, mean age 11.7 ± 2.7 years, 15 were male). Of these, 12 patients and 10 controls were scanned with single RF transmission while 29 patients and 12 controls were scanned in the PTx mode. One patient was excluded from the analysis because of widespread abnormalities due to extensive brain injury, which prevented clear identification of a focal lesion in either hemisphere and hindered reliable cortical segmentation. The clinical details of the patients recruited are summarized in eTable 1.

### Safety and Tolerability

No adverse events occurred during the 3T or 7T MRI scans. The 7T scan was generally well tolerated, with only mild and transient discomfort (related to neck pain or ear pain due to headphone position) reported in 38% of participants. More uncommon were dizziness, sensation of a metallic taste in the mouth, and body twitching, collectively reported in 25% of 7T scans.

When looking specifically at reports of dizziness, the McNemar χ^2^ test for dichotomized data indicated a statistically significant increase at 7T, observed in 55% of participants, compared with 9% at 3T (*p* = 0.02, Cohen *h* = 0.89, eTable 2). However, this effect was reported as temporary and only occurred while the patient was moving in and out of the scanner bore on the bed.

The most common reported discomfort associated with the scans was noise, which was defined as loud by approximately 67% of the population. There was no difference in noisiness, discomfort, metallic taste in the mouth, and body twitches between field strengths.

The Mann-Whitney test conducted to analyze differences between the groups aged 8–11 and 12–17 regarding 7T-associated effects showed that both noise and sensation of metallic taste in the mouth were more common in the younger group (noise: *p* = 0.02, *r* = −0.36; metallic taste: *p* = 0.02, *r* = −0.37, eTable 3).

### Quantitative Assessment of Cortical Thickness

The paired-sample *t* test revealed that the mean cortical thickness was significantly lower at 7T compared with 3T for both the right hemisphere (3T: mean = 3.06 mm; 7T: mean = 2.98 mm; mean difference = 0.0934 mm; 95% CI 0.0469–0.1399; *t* = 5.65, *p* < 0.001, Cohen *d* = −0.72) and the left hemisphere (3T: mean = 3.06 mm; 7T: mean = 2.96 mm; mean difference = 0.0934 mm; 95% CI 0.0469–0.1399; *t* = 5.01, *p* < 0.001, Cohen *d* = −0.64). The whole-brain vertex-wise comparison further revealed that the cortical thickness was reduced at 7T compared with 3T, particularly in temporo-parietal/sensory regions (Figure 2D).

### Radiologic Image Quality Assessment

Image quality was rated as very good for 3T images, with little or no artifacts reported in 90% of cases. At 7T, the MP2RAGE had little or no artifacts in 85% of cases, with moderate effects of motion in 15%. 7T FLAIR images in the scans evaluated for image quality were all acquired using single RF transmission. These images were all affected by a consistent signal loss in the right temporal lobe area due to the presence of B_1_ field inhomogeneities (Figure 2A): for this reason, 90% of images were scored as moderately artifacted, even if most of the cortex was of high quality. As shown in Figure 2, A and B, PTx significantly reduces signal loss in the right temporal lobe compared to single RF transmission. Ten percent of FLAIR images were also heavily affected by motion.

### Clinical Value of 7T MRI

**Figure 3:**
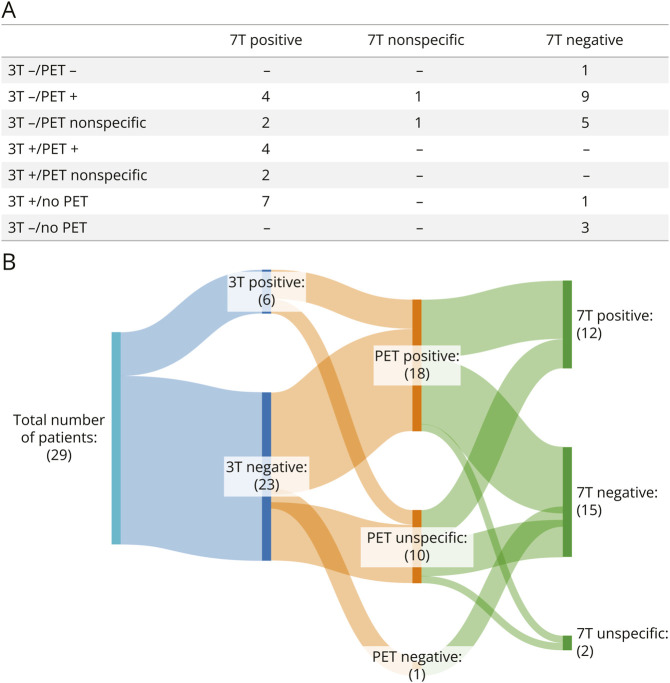
Lesion Detection Yield (A) Summary of findings across all patients included in the analysis (N = 40). (B) Sankey diagram of clinical outcomes in patients who underwent PET, 3T, and 7T imaging (N = 29).

The review of the images of the 40 patients with epilepsy showed that lesion detection increased from 35% (14/40) at 3T to 50% (20/40) at 7T (odds ratio 1.86). Figure 3 presents results for patients overall (A) and for patients who underwent all 3 (3T MRI, 7T MRI, PET) imaging modalities (B).

**Figure 4:**
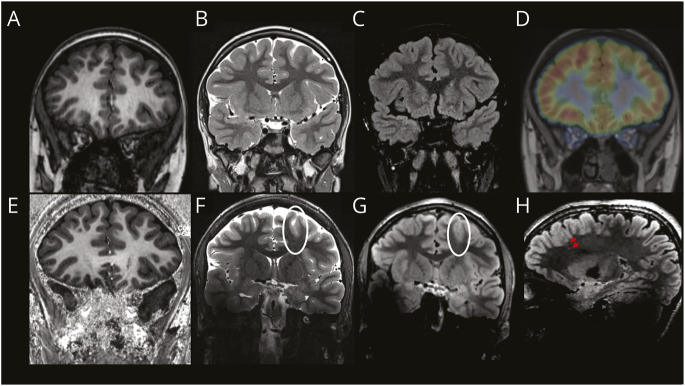
Case 1 (Patient 1) Patient with severe treatment-resistant frontal lobe epilepsy, with nocturnal hypermotor seizures up to 3 times per week. The EEG suggested a left frontal lobe origin of seizures. The 3T MRI (top row; A: MPRAGE, B: T2w, C: FLAIR) highlighted the presence of a deeper left superior frontal sulcus but without an obvious cortical thickening or signal change in the gray-white matter interface. A cortical lesion was not seen. However, the PET scan demonstrated heterogeneity of tracer uptake in the left frontal lobe compared with the right and a clear focal area of hypometabolism in the left superior frontal sulcus (clearly visible after PET + MR co-registration, top row, D). The 7T MP2RAGE (E) image did not show any abnormality, but the 7T T2w (F) and FLAIR (G, H) images displayed disruption of the gray-white matter boundary and the presence of a subtle transmantle sign. These findings matched the PET results and correlated well with EEG and semiology. An FCD was suspected, and because of the high conspicuity in an area of noneloquent cortex, it was decided that there was no need for a stereo-EEG and the patient underwent neurosurgery. The histologic finding was of an FCD type IIB. The patient was seizure free at 3 years of follow-up. FCD = focal cortical dysplasia; FLAIR = fluid-attenuated inversion recovery; MPRAGE = magnetization-prepared rapid gradient-echo; MP2RAGE = magnetization-prepared 2 rapid acquisition gradient-echo; T2w = T2-weighted.

**Figure 5:**
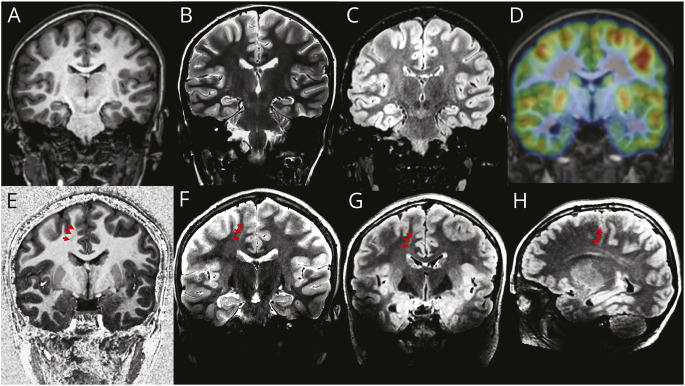
Case 2 (Patient 7) Patient affected by severe frontal lobe epilepsy, with nocturnal seizures (up to 20 episodes per night). The EEG suggested a right frontal lobe origin of seizures; however, the 3T MRI (top row; A: MPRAGE; B: T2w; C: FLAIR) did not show a convincing lesion. The PET scan (D) detected a reduced tracer uptake in the right postcentral sulcus and right frontotemporal operculum. The 7T MRI (bottom row; E: MP2RAGE; F: T2w; G, H: FLAIR) highlighted a structural abnormality originating in the right frontal lobe: a subtle hyperintense signal track was seen extending from the medial frontal lobe toward the lateral ventricle, associated with blurring of the gray-white matter interface. This new information helped with restricting the area under investigation during stereo-EEG. The patient subsequently underwent surgery, and histologic examination confirmed the presence of an FCD type IIB. The patient was seizure free at 3 years of follow-up. FCD = focal cortical dysplasia; FLAIR = fluid-attenuated inversion recovery; MPRAGE = magnetization-prepared rapid gradient-echo; MP2RAGE = magnetization-prepared 2 rapid acquisition gradient-echo; T2w = T2-weighted.

**Figure 6:**
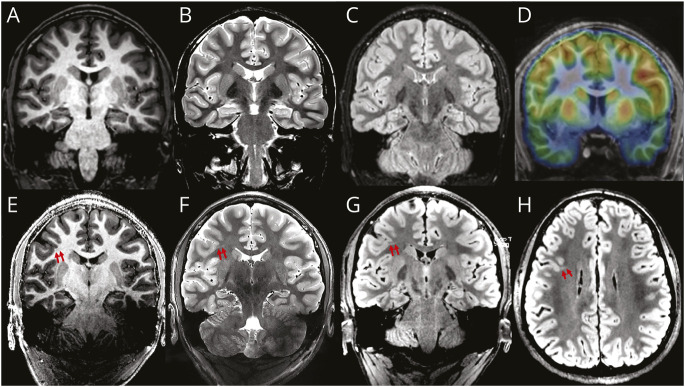
Case 3 (Patient 18) Patient affected by predominantly nocturnal seizures (up to 4/5 times per week). The video-EEG captured seizures with electrical correlate from the right frontal region; however, the 3T MRI (top row; A: MPRAGE; B: T2w; C: FLAIR) was reported as normal. The PET scan (D) detected a very focal reduced tracer uptake in the right inferior frontal sulcus. The 7T MRI (bottom row; E: MP2RAGE; F: T2w; G, H: FLAIR) highlighted a faint band of mildly increased signal in the FLAIR image extending into the white matter from the deep cortical margin in the depth of the right inferior frontal sulcus posteriorly (transmantle sign). This was detected precisely in the same sulcus as seen in the PET-MRI co-registration. The stereo-EEG confirmed epileptic activity originating from that area. A thermocoagulation procedure was undertaken, and the patient was seizure free at 2 years of follow-up. FCD = focal cortical dysplasia; FLAIR = fluid-attenuated inversion recovery; MPRAGE = magnetization-prepared rapid gradient-echo; MP2RAGE = magnetization-prepared 2 rapid acquisition gradient-echo; T2w = T2-weighted.

Specifically, 8 of 26 patients who were lesion-negative at 3T had new findings at 7T. In 2 patients, these findings were nonspecific, including ventricular asymmetry with a more prominent left occipital horn (patient 2, 14 years old) and an asymmetrically deep sulcus in the left superior frontal gyrus (patient 24, 9 years old). In the remaining 6 patients, new lesions were identified at 7T. Specifically, patients 1 (13 years old, left frontal lesion, Figure 4), 5 (13 years old, left temporal lesion, eFigure 1), 7 (10 years old, right frontal lesion, Figure 5), 18 (15 years old, right frontal lesion, Figure 6), 34 (13 years old, left temporal lesion, eFigure 2), and 41 (16 years old, left frontal lesion, eFigure 3) had new specific findings reported at 7T.

Of these, 6 (100%) had PET findings (4/6 PET findings showed concordant localizing features while 2/6 findings were not localizing). Reviewing the first 5 cases that were 3T lesion-negative but lesion-positive at 7T, radiologists later confirmed that the lesion was retrospectively visible at 3T but was either ambiguous or less conspicuous compared with 7T.

Of the 6 patients with new specific findings at 7T, 2 (patient 1 and patient 7) underwent surgical resection and both were seizure free at 3 years of follow-up. Owing to the 7T findings, stereo-EEG (SEEG) was re-planned with a reduced number of electrodes in one patient and omitted in the other. In another patient (patient 18), the SEEG demonstrated a focal onset of seizures in the putative lesion seen on the 7T MRI that was concordant with hypometabolism seen on the PET scan. Radiofrequency thermocoagulation was performed, and the patient was seizure free at 2 years of follow-up. These 3 cases are presented in detail in Figures 4–6. For a fourth patient (patient 36), the 7T images helped with SEEG implantation and to better identify lesion margins. The lesion area detected with 7T MRI was consistent with the area detected with SEEG. For the remaining patients, the 7T scan findings did not affect clinical decision making. Detailed descriptions of these cases are presented in Figures 4–6 while eTable 1 summarizes clinical outcomes for all patients.

In patients who were lesion-positive at 3T (n = 14), findings were replicated at 7T in all but 1 patient (patient 38, eFigure 4) (93%). For this patient, potential polymicrogyria in the right middle frontal gyrus were suspected at 3T, in concordance with the clinical EEG localization. This finding was not confirmed at 7T.

There was 1 incidental finding at 7T in a 3T lesion-negative epileptic patient: a small cyst in the subcortical white matter of no clinical significance and unrelated to epilepsy. There were no new incidental findings at 7T MRI in the healthy control population.

## Discussion

We systematically evaluated image quality, scan tolerability, and clinical utility of 7T MRI scanning in children with focal epilepsy aged 8 years and older. Our findings suggest that 7T MRI is both feasible and well tolerated in this age group and holds significant potential for enhancing lesion detection and supporting presurgical planning in focal epilepsy cases where conventional MRI results are inconclusive.

A key factor in understanding the practical utility of 7T MRI in children is to characterize the type and frequency of its associated side effects. Previous adult studies have shown that short-term effects such as dizziness, vertigo, nausea, headache, and a metallic taste may be experienced by participants during ultra-high field scanning and that reported side effects are larger than for lower field strengths.^23,24,39-42^ In line with this, our pediatric study demonstrated that increased dizziness was experienced during 7T scanning in comparison with 3T scanning. However, this settled after the participant had stopped moving through the field; none of the reported side effects persisted beyond the scanning procedure, and no adverse events were observed during the scanning session. Notably, side effects were more commonly reported among younger children, suggesting that the experience of higher field might differ based on age-related factors and developmental stages. This underscores the importance of tailoring procedures and providing adequate support and reassurance, especially for younger children.

Next, we investigated quantitative measures of image quality. Specifically, we compared cortical thickness measurements at 3T and 7T as a quantitative measure of a clinically relevant image characteristic. Our findings demonstrated thinner cortical boundaries at 7T, particularly in temporo-parietal/sensory regions. These results align with evidence from a previous study in adult participants^43^ and suggest improved gray matter segmentation due to higher resolution and better contrast at 7T. Though an indirect measure (and not a ground truth of cortical thickness in the brain), the metric is a good proxy for gray/white matter boundary sharpness, the breakdown of which is a subtle marker of some cortical dysplasias.

Finally, clinical research in adults has demonstrated the sensitivity and clinical value of 7T MRI in patients with drug-resistant focal epilepsy, with a notable improvement in the detection rate of epileptogenic lesions ranging between 20% and 30% in those considered MRI negative at 3T.^18-21^ In this study, 7T MRI revealed new subtle lesions in 23% of children with previously unrevealing 3T scans, mirroring the rates reported in adult populations. There was no clear difference in age between patients who had new specific findings at 7T (mean age 13.3 years) and those who remained negative at both 3T and 7T (mean age 12.4 years), suggesting that lesion detectability at 7T was not related to age in this sample. Similarly, new lesion detection at 7T did not show a clear bias toward a specific brain region, with findings distributed evenly across the left frontal (33%), left temporal (33%), and right frontal (33%) lobes. This underscores the potential of 7T as a valuable tool for improved diagnostic outcomes in pediatric focal epilepsy management.

These results should be considered a baseline. Wang et al.^38^ highlighted the importance of postprocessing approaches in increasing total lesion detection yield. Specifically, their study demonstrated that while unaided visual review alone generated 22% yield in 3T MRI–negative patients, the addition of postprocessing methods markedly increased yield to 43%. In addition, the application of machine learning algorithms shows further promise in enhancing lesion detection in focal epilepsy. For example, the Multi-centre Epilepsy Lesion Detection Project^44^ demonstrated enhanced detection and classification of lesions in patients with focal epilepsy using an AI-driven approach, achieving a detection rate of 70% overall and 62% in MRI-negative cases.

In this study, no new findings were reported in 3T MRI–positive patients and typically developing controls. However, in 1 patient, potential polymicrogyria in the right middle frontal gyrus were suspected at 3T, in concordance with the clinical EEG localization. With the higher resolution at 7T, this was ruled out. This discrepancy underscores the potential of 7T MRI's higher resolution and sensitivity in potentially reducing false positives.^38^ Although it is essential to consider other factors such as imaging artifacts, which could have contributed to the contrasting results, no artifacts were reported in this patient's scan.

Two of the 6 patients with new findings at 7T have undergone surgical resection so far, and FCD type IIb was confirmed by histopathology for both. Because of the 7T findings confirming the PET-MR co-registration findings in these previously 3T-negative cases, SEEG was re-planned with a reduced number of electrodes in 1 patient and omitted in the other. At 3 years after surgery, both patients were seizure free. Another patient has undergone successful thermocoagulation after a subtle lesion was identified at 7T, confirming the PET-MR co-registration result, and was seizure free at 2 years of follow-up. In a fourth patient, the 7T scan helped with SEEG implantation and to better identify lesion margins. 7T may, therefore, be particularly useful when expert PET-MR co-registration is not available and has the potential of avoiding SEEG in selected cases.

This study has some limitations. First, patients had been extensively investigated in the Children's Epilepsy Surgery Service to precisely define the seizure-onset zone and decide patients' management. This could have increased the probability of detecting structural abnormalities in our cohort. Second, 3T motion-corrected images were compared with 7T uncorrected images. In examinations affected by motion, this might have prevented the visualization of a structural lesion at 7T. Third, not all patients had the 7T FLAIR acquired with parallel transmit, because this had not yet been implemented when the study started. Three of 6 patients (50%) with new lesions at 7T (patients 18, 24, 41) were scanned with single transmit, indicating that improved detection at 7T is not solely due to parallel transmit. However, because none of these lesions was in the right temporal lobe, B1 field inhomogeneities—particularly in the anterobasal right temporal region—may have affected lesion detection in some cases. Although assessing the potential added benefit of PTx at 7T was beyond the scope of this study, we plan to address this in a future study.

Despite these limitations, our findings suggest that 7T MRI in children and adolescents is feasible, well tolerated, and associated with increased lesion detection in patients with focal epilepsy with unrevealing conventional MRI. 7T MRI may play an important role in facilitating presurgical planning and permitting a more focused and less invasive approach in these patients, leading to a better surgical outcome.

Although technically more challenging to visualize, the improved SNR and spatial resolution at 7T may also offer the potential to better detect structural abnormalities near the skull base. While field inhomogeneities and susceptibility artifacts do pose challenges in areas adjacent to bone and air-filled cavities, these can be mitigated using PTx and B1+ insensitive protocols and dielectric pads,^17^ enhancing visualization. Notably, recent studies suggest that 7T MRI may be superior at detecting pathology in the skull base.^45^

It is also worth noting that the applicability of 7T MRI is limited by safety considerations, particularly for patients with implants such as vagus nerve stimulators, deep brain stimulators, or metallic clips. Nevertheless, while these constraints are particularly relevant for adults with epilepsy, they are less applicable for pediatric patients, in whom implanted devices are less common.

Overall, future advancement in MR technology, such as the integration of motion correction and the utilization of advanced computational postprocessing methods, has the potential to enhance the clinical value of 7T imaging in detecting structural brain lesions.^17^ In addition, given the higher resolution and increased data volume produced by 7T MRI, any assistance in streamlining visual review processes is likely to yield substantial advantages. In particular, co-registration of PET and high-resolution MRI can provide an important prior to a focal hypothesis, thus facilitating the identification of the epileptogenic onset zone.^14^ Accordingly, in this study, 6 of 6 3T MRI–negative patients (100%) with new specific findings at 7T had previous PET findings.

Generally, this also highlights the importance of careful and appropriate selection of patients who are likely to benefit from a 7T investigation. Specifically, patients for whom a very specific preimaging hypothesis has been derived, from a combination of clinical and imaging data, may be the most likely to benefit from an ultra-high resolution scan at 7T.

## Glossary

DISORDER: distributed and incoherent sample orders for reconstruction deblurring using encoding redundancy
FCD: focal cortical dysplasia
FLAIR: fluid-attenuated inversion recovery
HCP: Human Connectome Project
MPRAGE: magnetization-prepared rapid gradient-echo
MP2RAGE: magnetization-prepared 2 rapid acquisition gradient-echo
PTx: parallel RF transmission
RF: radiofrequency
SEEG: stereo-EEG
SNR: signal-to-noise ratio
